# Comparative risk evaluation for cardiovascular events associated with dapagliflozin vs. empagliflozin in real-world type 2 diabetes patients: a multi-institutional cohort study

**DOI:** 10.1186/s12933-019-0919-9

**Published:** 2019-09-24

**Authors:** Shih-Chieh Shao, Kai-Cheng Chang, Ming-Jui Hung, Ning-I Yang, Yuk-Ying Chan, Hui-Yu Chen, Yea-Huei Kao Yang, Edward Chia-Cheng Lai

**Affiliations:** 1 Department of Pharmacy, Keelung Chang Gung Memorial Hospital, Keelung, Taiwan; 2 School of Pharmacy, Institute of Clinical Pharmacy and Pharmaceutical Sciences, College of Medicine, National Cheng Kung University, No. 1, University Road, Tainan, 701 Taiwan; 3 Department of Pharmacy, Linkou Chang Gung Memorial Hospital, Taoyuan, Taiwan; 4 Division of Cardiology, Department of Internal Medicine, Chang Gung Memorial Hospital, Keelung, Taiwan; 5 Chang Gung University College of Medicine, Taoyuan, Taiwan; 6 Department of Pharmaceutical Materials Management, Chang Gung Medical Foundation, Taoyuan, Taiwan

**Keywords:** Sodium–glucose co-transporter 2 inhibitors, Cardiovascular disease, Heart failure, Real-world data

## Abstract

**Background:**

To compare the cardiovascular event risk in type 2 diabetes patients newly receiving dapagliflozin vs. empagliflozin.

**Methods:**

We conducted a retrospective cohort study by analyzing a multi-institutional electronic medical records database (Chang Gung Research Database) in Taiwan and included adult type 2 diabetes patients who were newly receiving sodium–glucose co-transporter 2 (SGLT2) inhibitors from 2016 to 2017. The primary outcome was a composite of cardiovascular death, myocardial infarction, ischemic stroke and heart failure. We followed up patients from initiation of SGLT2 inhibitors until the occurrence of cardiovascular events before December 31, 2018. We performed multivariable Cox proportional hazard modeling, adjusting for patients’ age, sex, laboratory data, co-morbidities, and concomitant medications.

**Results:**

We identified 12,681 new SGLT2 inhibitor users with a mean age of 58.9 (SD 11.8) years, of whom 43.9% were female and 45.8% were new dapagliflozin users. A total of 10,442 person-years of dapagliflozin use and 12,096 person-years of empagliflozin use were included. Compared to empagliflozin users, new users of dapagliflozin were found to have similar risks for primary composite outcome (adjusted HR: 0.91; 95% CI 0.73–1.14), cardiovascular death (adjusted HR: 0.54; 95% CI 0.14–2.12), myocardial infarction (adjusted HR: 0.77, 95% CI 0.49–1.19) and ischemic stroke (adjusted HR: 1.15; 95% CI 0.80–1.65), but a lower risk of heart failure (adjusted HR: 0.68; 95% CI 0.49–0.95).

**Conclusion:**

The risk of cardiovascular events was similar between dapagliflozin and empagliflozin new users, but dapagliflozin may have a better outcome in the reduction of heart failure in type 2 diabetes patients. Future prospective studies are required to confirm the findings.

## Background

Patients with type 2 diabetes suffer substantial morbidity and mortality from cardiovascular disease [1]. Current medication and lifestyle interventions may not be sufficient to reduce the risk of serious cardiovascular disease outcomes in the primary prevention cohorts of type 2 diabetes [2]. Although the largest absolute benefits of interventions for individual patients are achieved among those with established atherosclerotic cardiovascular disease, the large number of type 2 diabetes patients without a history of major cardiovascular disease makes knowledge about the effects of anti-diabetes medication on first events an additional priority [2].

Sodium–glucose co-transporter 2 (SGLT2) inhibitor which decreases glucose re-absorption in the kidney and increases excretion via the urine is the new drug class for type 2 diabetes management with favorable safety profiles [3, 4], and is recommended as an option for treatment intensification after the failure of metformin [5]. A meta-analysis of three large placebo-controlled cardiovascular outcome trials found that SGLT2 inhibitors reduced major adverse cardiovascular events by 11% (HR: 0.89, 95% CI 0.83–0.96). In addition, compared to incretin-based therapies, including glucagon-like-peptide 1 (GLP-1) agonists and dipeptidyl peptidase 4 (DPP-4) inhibitors, SGLT2 inhibitors are associated with lower risks of all-cause and cardiovascular-specific mortality, and occurrence of heart failure and myocardial infarction [6]. Although the direct cardioprotective mechanisms are not fully understood, they may be related to hemodynamic and metabolic actions from SGLT2 inhibitors [7–9].

SGLT2 inhibitors, in addition to glycemic controls, also have a positive effect on the cardiometabolic markers, such as body weight, blood pressure and uric acid [10, 11], and as a result there has been increasing use of SGLT2 inhibitors for the management of type 2 diabetes in clinical practice [12]. However, not all SGLT2 inhibitors share the same pharmacokinetic properties; for example, dapagliflozin with its slower excretion by the kidney exerts its pharmacological effects longer, even after 18 h post-dose, whereas the effects of empagliflozin are markedly attenuated from 12 h post-dose [13]. Due to more stable and longer sodium excretion and osmotic diuresis effects when compared to empagliflozin, dapagliflozin has been reported to reduce the 24-h variability in systolic blood pressures and may be associated with a lower risk for cardiovascular diseases [14–17].

Current evidence has shown the beneficial effects of SGLT2 inhibitors on cardiovascular events, but the effects may differ between the individual SGLT2 inhibitors. Therefore, the purpose of this study was to determine the comparative cardiovascular event risk associated with dapagliflozin vs. empagliflozin in real-world type 2 diabetes patients.

## Methods

### Data source

This retrospective cohort study analyzed data from Chang Gung Research Database (CGRD), the largest multi-institutional electronic medical records database in Taiwan [18]. The CGRD was established for research purposes in 2016, and currently covers 1.3 million patients (6% of the population of Taiwan). The CGRD includes clinical data for all patients who had outpatient treatment or were hospitalized in one of the 7 Chang Gung Memorial Hospitals from northern to southern Taiwan. The CGRD identifies diseases based on the International Classification of Diseases, Ninth Revision, Clinical Modification (ICD-9-CM) before 2016, and ICD-10-CM afterwards. Unlike administrative data, the CGRD contains laboratory data which can provide a valid estimate of association between outcomes and exposures. The structures of CGRD have been described in another publication [18], and the accuracy and validity of the diagnostic codes in CGRD are well established [19–21].

### Study population

From the CGRD we identified type 2 diabetes patients newly treated with a SGLT2 inhibitor, either dapagliflozin or empagliflozin, during 2016–2017. The index date was defined as the date of SGLT2 inhibitor initiation. Because SGLT2 inhibitors were approved for type 2 diabetes management starting in 2016, all patients in this study could be considered as new users. We defined the baseline period as 1 year prior to the index date. Patients were excluded if they were less than 18 years old. Patients also needed to have had at least one clinical visit before the index date, in order to establish their baseline information. To ensure that cardiovascular events represented new cases, we excluded patients who had diagnoses of myocardial infarction, ischemic stroke and heart failure within 1 year prior to the index date. We excluded patients without laboratory examinations including body mass index (BMI), blood sugar controls (e.g., HbA1c), renal functions (e.g., estimated glomerular filtration rate; eGFR and urine albumin–creatinine ratio; UACR) or lipid profiles (e.g., low-density lipoprotein; LDL) before the index date, because these patients may not receive routine medical care in our hospitals. Finally, we also excluded patients who incurred cardiovascular events of interest within 30 days after the index date, considering that such a short-term exposure to SGLT2 inhibitors was unlikely to be the cause of cardiovascular events.

### Outcomes definition and follow-up

The primary outcome was the composite event of cardiovascular mortality, myocardial infarction, ischemic stroke and heart failure in the diagnosis of hospitalization and outpatient data. As secondary outcomes we evaluated the risk of the aforementioned individual events. Although studies have indicated that these cardiovascular outcome diagnosis codes have high positive predictive values (91.5–97.9%) in Taiwan’s healthcare settings [20, 22, 23], we also reviewed the electronic medical records from 3 Chang Gung Memorial Hospitals to assess the validity of the diagnosis codes corresponding to the cardiovascular events of interest. Specifically, we confirmed the heart failure diagnoses by laboratory examination data (e.g., B-type Natriuretic Peptide, BNP), left ventricular ejection fraction (LVEF) from echocardiogram as well as the symptoms of heart failure (e.g., dyspnea). We reviewed the medical charts in 222 cardiovascular events, and found the positive predictive values of 75.0%, 95.8%, 91.9% and 89.8% for cardiovascular mortality, myocardial infarction, ischemic stroke and heart failure, respectively. We performed intention-to-treat analysis and followed patients until the occurrence of the cardiovascular outcomes, death or last date of clinical records in the CGRD or December 31, 2018.

### Covariates

Approximately 40 potential confounding covariates based on previous studies and experts’ opinions were included in the regression models [24, 25]. We collected data on individual co-morbidities (i.e., hypertension and dyslipidemia, coronary heart diseases and microvascular complications) and computed composite scores of co-morbidities (i.e., Charlson comorbidity index [26]) at baseline. We also collected data of concomitant medications (i.e., statin, antiplatelet agents, anticoagulant agents, antihypertensive agents and antihyperglycemia agents) within 3 months before the index date. We included the most recent laboratory data before SGLT2 inhibitor initiation to evaluate the baseline cardiovascular risk. For the missing values of laboratory data, we performed multiple imputations using the Markov chain Monte Carlo method with a combination of 10 simulations [27, 28]. The details of co-morbidity and concomitant medications are described in Additional file 1: Table S1, Additional file 2: Table S2.

### Negative control outcome

Negative control outcome could detect residual confounding and bias due to unobserved confounders [29, 30]. We examined the association of SGLT2 inhibitors with incident atrial fibrillation as a negative control outcome in this study population. Because there is currently no plausible mechanism by which SGLT2 inhibitors are associated with an altered risk of atrial fibrillation [25], any observed significant associations in this falsification test should be due to bias.

### Statistical analysis

We defined the patients with empagliflozin as the reference group, and used multivariable Cox proportional hazards models to estimate the hazard ratios (HR) and 95% confidence intervals (CI) of outcomes. The variables used in the Cox proportional hazards models included age, sex, laboratory data, co-morbidities and concomitant medications (Table 1). All variables significantly related to outcomes from the prior uni-variate Cox regression model at alpha level of 0.1 were included by stepwise selection in the multivariate analyses. All analyses were two-tailed with a type I error level at 0.05, and were conducted using SAS Enterprise (Version 5.1; SAS Institute Inc., Cary, NC, USA).

**Table 1:** Baseline patient characteristics

	Dapagliflozin	Empagliflozin	P value
Patients, n (%)	5812 (45.8)	6869 (54.2)	
Age, n (%), years			< 0.001
< 65	4041 (69.5)	4497 (65.5)	
≧ 65	1771 (30.5)	2372 (34.5)	
Female, n (%)	2647 (45.5)	2920 (42.5)	< 0.001
PDD/DDD, mean (SD)^a^	0.9 (0.2)	0.8 (0.4)	< 0.001
Prescribed daily dose, n (SD)^b^			< 0.001
Low dose, n (%)	1451 (25.0)	4071 (59.3)	
Full dose, n (%)	4361 (75.0)	2798 (40.7)	
BMI, n (%), kg/m^2^			0.576
< 30	3905 (67.2)	4583 (66.7)	
≥ 30	1907 (32.8)	2286 (33.3)	
HbA1c, n (%), %			< 0.001
< 8.5	2541 (43.7)	3308 (48.2)	
≥ 8.5	3271 (56.3)	3561 (51.8)	
eGFR, n (%), mL/min/1.73 m^2^			< 0.001
< 60	347 (6.0)	930 (13.6)	
60–90	2102 (36.2)	2473 (36.0)	
≥ 90	3363 (57.9)	3466 (50.5)	
UACR, n (%), mg/g			< 0.001
< 30	3007 (51.7)	3336 (48.6)	
30–300	1834 (31.6)	2199 (32.0)	
≥ 300	971 (16.7)	1334 (19.4)	
LDL, n (%), mg/dL			0.505
≥ 100	104 (1.8)	134 (2.0)	
< 100	5708 (98.2)	6735 (98.0)	
Index year, n (%)			< 0.001
2016	3326 (57.2)	3624 (52.8)	
2017	2486 (42.8)	3245 (47.2)	
Hospital level, n (%)			< 0.001
Medical centers	3224 (55.5)	3927 (57.2)	
Regional hospitals	1540 (26.5)	1533 (22.3)	
District hospitals	1048 (18.0)	1409 (20.5)	
Department, n (%)			< 0.001
Metabolism and endocrinology	3964 (68.2)	4206 (61.2)	
Cardiology	1181 (20.3)	1792 (26.1)	
Others	667 (11.5)	871 (12.7)	
Comorbidity, n (%)			
Hypertension	3718 (64.0)	4606 (67.1)	< 0.001
Hyperlipidemia	4274 (73.5)	4974 (72.4)	0.155
Coronary heart disease^c^	832 (14.3)	1207 (17.6)	< 0.001
Atrial fibrillation	113 (1.9)	166 (2.4)	0.071
Peripheral artery disease	67 (1.2)	108 (1.6)	0.044
Diabetic retinopathy	475 (8.2)	577 (8.4)	0.644
Diabetic neuropathy	583 (10.0)	627 (9.1)	0.085
Diabetic nephropathy	1510 (26.0)	1893 (27.6)	0.046
Chronic obstructive pulmonary disease	126 (2.2)	177 (2.6)	0.133
Liver disease	1052 (18.1)	1278 (18.6)	0.465
Depression	85 (1.5)	102 (1.5)	0.917
Schizophrenia	24 (0.4)	19 (0.3)	0.188
Cancer	366 (6.3)	445 (6.5)	0.678
Charlson comorbidity index score, n (%)			0.007
< 2	2440 (42.0)	2722 (39.6)	
≥ 2	3372 (58.0)	4147 (60.4)	
Previous hospitalization, n (%)	554 (9.5)	815 (11.9)	< 0.001
Concomitant medications, n (%)			
Anti-platelet agents	1549 (26.7)	2074 (30.2)	< 0.001
Anti-coagulant agents	100 (1.7)	151 (2.2)	0.054
Beta blockers	1249 (21.5)	1690 (24.6)	< 0.001
Angiotensin-converting enzyme inhibitors or angiotensin receptor blockers	3235 (55.7)	4129 (60.1)	< 0.001
Calcium channel blockers	2056 (35.4)	2775 (40.4)	< 0.001
Loop diuretics	153 (2.6)	282 (4.1)	< 0.001
Thiazides	270 (4.7)	342 (5.0)	0.383
Mineralocorticoid receptor antagonist	59 (1.0)	124 (1.8)	< 0.001
Statin	3662 (63.0)	4538 (66.1)	< 0.001
Fibrate	535 (9.2)	717 (10.4)	0.020
Ezetimibe	646 (11.1)	793 (11.5)	0.447
Metformin	5364 (92.3)	6124 (89.2)	< 0.001
Sulfonylurea	3604 (62.0)	3751 (54.6)	< 0.001
Dipeptidyl peptidase-4 inhibitors	3616 (62.2)	4437 (64.6)	0.006
Alpha-glucosidase inhibitors	1002 (17.2)	1240 (18.1)	0.232
Glinides	94 (1.6)	158 (2.3)	0.006
Thiazolidinediones	1552 (26.7)	1584 (23.1)	< 0.001
Glucagon-like peptide-1 receptors antagonist	75 (1.3)	156 (2.3)	< 0.001
Insulin	1083 (18.6)	1507 (21.9)	< 0.001
Non-steroidal anti-inflammatory drugs	475 (8.2)	542 (7.9)	0.560

*BMI* body mass index, *DDD* defined daily dose, *eGFR* estimated glomerular filtration rate, *LDL* low-density lipoprotein, *PDD* prescribed daily dose, *UACR* urine albumin–creatinine ratio

^a^The ratio of prescribed daily dose (PDD)/defined daily dose (DDD) is defined by the WHO Collaborating Center (https://www.whocc.no/atc_ddd_index/) for drug comparisons [62]; that is, the PDD/DDD ratio of patients who used 10 mg dapagliflozin daily is equal to 17.5 mg empagliflozin (PDD/DDD ratio = 1)

^b^Low dose: dapagliflozin < 10 mg and empagliflozin < 25 mg; full dose: dapagliflozin 10 mg and empagliflozin 25 mg

^c^Myocardial infarction was not included because prevalent myocardial infarction cases were excluded before initiation of SGLT2 inhibitors in this study

### Sensitivity and subgroup analyses

We performed several sensitivity and subgroup analyses to create more homogenous groups for comparisons to test robustness of results from the main analyses. First, to evaluate the effects from non-adherence to the SGLT2 inhibitors, we performed per-protocol design and followed patients until the occurrence of cardiovascular outcome or discontinuation of the initial SGLT2 inhibitors (e.g., grace period over 90 days or switching to another SGLT2 inhibitor), whichever came first. Second, because patients who died from causes other than cardiovascular events (e.g., cancer) were no longer at risk of the study outcomes, this non-cardiovascular mortality was regarded as the major competing risk in the analyses. To address the possible competing risk events, we specifically compared rates of non-cardiovascular mortality between the empagliflozin and dapagliflozin groups. We found the rates of mortality were similar between the two groups (5.0 vs. 4.5 per 1000 person-years for empagliflozin vs. dapagliflozin, respectively), which implied that the effect of competing risk was minor. Nevertheless, we used the proportional subdistribution hazard models raised by Fine and Gray et al. [31] to manage possible competing risk of mortality. Third, we used three propensity score methods, including propensity score matching, stabilized inverse probability of treatment weighting (SIPTW) and standardized mortality ratio weighting (SMRW), to balance potential baseline differences between the empagliflozin and dapagliflozin groups. We describe patient characteristics before and after propensity score methods in Additional file 3: Table S3. Fourth, we increased washout periods up to 5 years for selection of patients without cardiovascular diseases to test the original analyses. Fifth, we excluded patients who incurred cardiovascular events of interest up to 1 year after the index date to avoid protopathic bias. Sixth, we repeated the analyses based on the specific SGLT2 inhibitor dose (i.e., low dose: dapagliflozin < 10 mg and empagliflozin < 25 mg; full dose: dapagliflozin 10 mg and empagliflozin 25 mg). Additionally, we conducted stratification analyses based on baseline risks for cardiovascular diseases, such as patients’ BMI levels (≥ 30 or < 30 kg/m^2^), blood sugar levels (HbA1c: ≥ 8.5 or < 8.5%), renal functions (eGFR: ≥ 90 or < 90 mL/min/1.73 m^2^; UACR: < 30 or ≥ 30 mg/g) and lipid profiles (LDL: ≥ 100 or < 100 mg/dL). Finally, we conducted additional analyses based on the specific concomitant medications which may modify the cardiovascular disease risk, including anti-platelet drugs, angiotensin-converting enzyme inhibitors (ACEI)/angiotensin receptor blockers (ARB) and loop diuretics.

## Results

### Study population

**Fig. 1:**
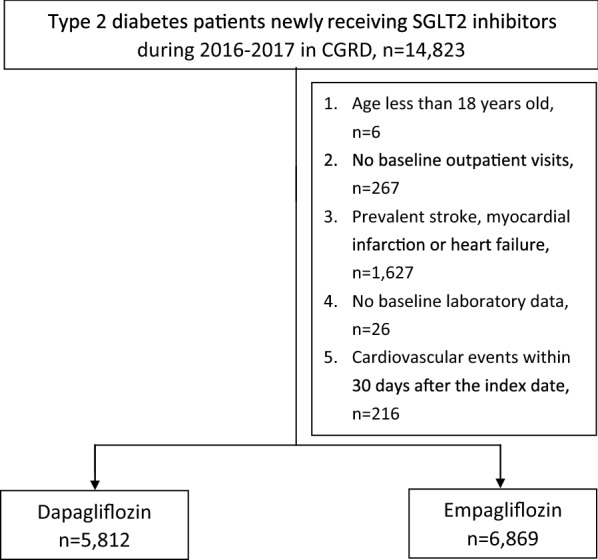
Patient selection flow chart. *CGRD* Chang Gung Research Database

We included a total of 12,681 patients newly initiating SGLT2 inhibitors, of which 5812 received dapagliflozin and 6869 received empagliflozin (Fig. 1). Patients newly receiving empagliflozin had worse renal functions at baseline (e.g., eGFR: 93.0 ± 31.6 vs. 97.7 ± 28.6 mL/min/1.73 m^2^; UACR: 242.9 ± 723.3 vs. 171.5 ± 502.7 mg/g). The mean baseline HbA1c levels (8.9 ± 1.6 vs. 8.8 ± 1.7%) and LDL levels (81.8 ± 23.0 vs. 81.5 ± 23.0 mg/dL) were comparable between dapagliflozin and empagliflozin new users. There were similar rates of co-morbidity between the SGLT2 inhibitors, but patients with empagliflozin had higher rates of hypertension (67.1% vs. 64.0%) and coronary heart disease (17.6% vs. 14.3%) at baseline. Greater use of anti-platelet drugs (30.2% vs. 26.7%), ACEI/ARB (60.1% vs. 55.7%), beta-blockers (24.6% vs. 21.5%), calcium channel blockers (40.4% vs. 35.4%) and loop diuretics (4.1% vs. 2.6%) was found in patients initiating empagliflozin than in those initiating dapagliflozin (Table 1).

### SGLT2 inhibitors and composite CV outcomes

We followed a total of 10,442 and 12,096 person-years with mean follow-up time of 656 days and 643 days for the dapagliflozin and empagliflozin group, respectively. There were 128 and 197 composite cardiovascular events in the dapagliflozin (incidence rate: 12.3 per 1000 person-years) and empagliflozin (incidence rate: 16.3 per 1000 person-years) groups, respectively. We found a similar risk of composite cardiovascular outcome (adjusted HR: 0.91; 95% CI 0.73–1.14), cardiovascular mortality (adjusted HR: 0.54; 95% CI 0.14–2.12), myocardial infarction (adjusted HR: 0.77; 95% CI 0.49–1.19) and ischemic stroke (adjusted HR: 1.15; 95% CI 0.80–1.65) between dapagliflozin and empagliflozin (Table 2). However, dapagliflozin use was associated with a lower risk of heart failure than empagliflozin (adjusted HR: 0.68; 95% CI 0.49–0.95).

**Table 2:** Comparative risk for cardiovascular events between dapagliflozin and empagliflozin (reference)

	Dapagliflozin, n (incidence rate)^a^	Empagliflozin, n (incidence rate)^a^	Crude HR	Adjusted HR
Composite outcome	128 (12.3)	197 (16.3)	0.75 (0.60–0.93)	0.91 (0.73–1.14)
Specific outcome				
Cardiovascular mortality	3 (0.3)	7 (0.6)	0.46 (0.12–1.76)	0.54 (0.14–2.12)
Myocardial infarction	33 (3.1)	53 (4.3)	0.70 (0.46–1.09)	0.77 (0.49–1.19)
Ischemic stroke	56 (5.3)	63 (5.2)	1.03 (0.72–1.48)	1.15 (0.80–1.65)
Heart failure	52 (4.9)	109 (9.0)	0.55 (0.39–0.76)	0.68 (0.49–0.95)

^a^Incidence rate was calculated by 1000 person-years

### Negative control outcome

In falsification testing, there were neutral associations between SGLT2 inhibitors and incident atrial fibrillation (adjusted HR: 1.08; 95% CI 0.73–1.60) (Additional file 4: Table S4).

### Sensitivity and subgroup analyses

**Fig. 2:**
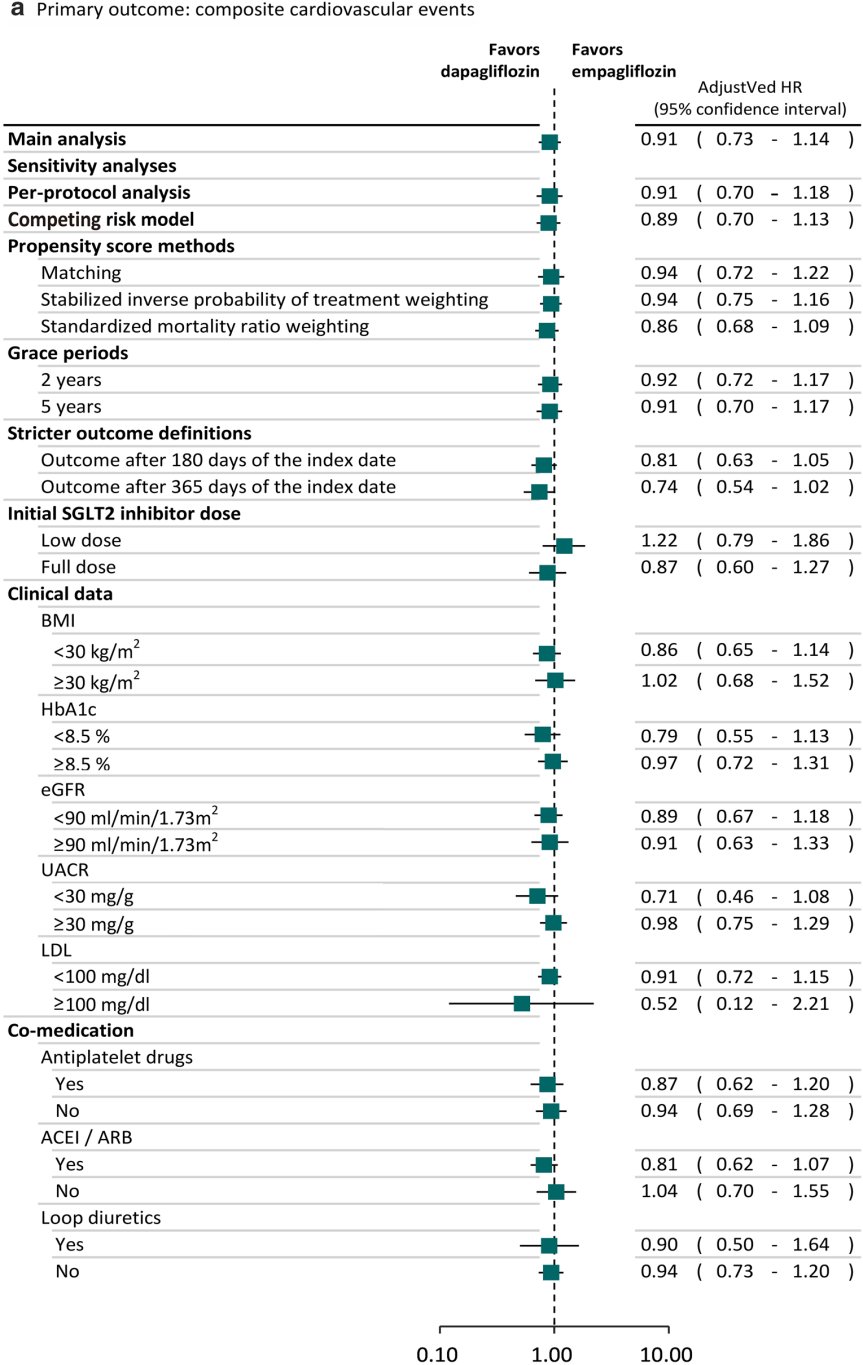

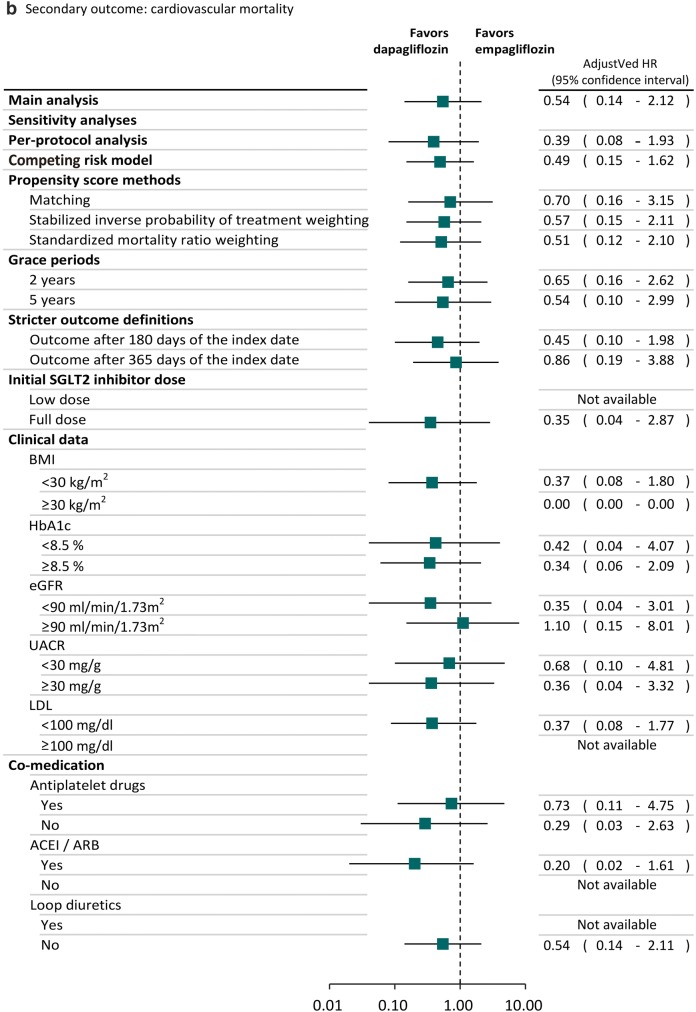

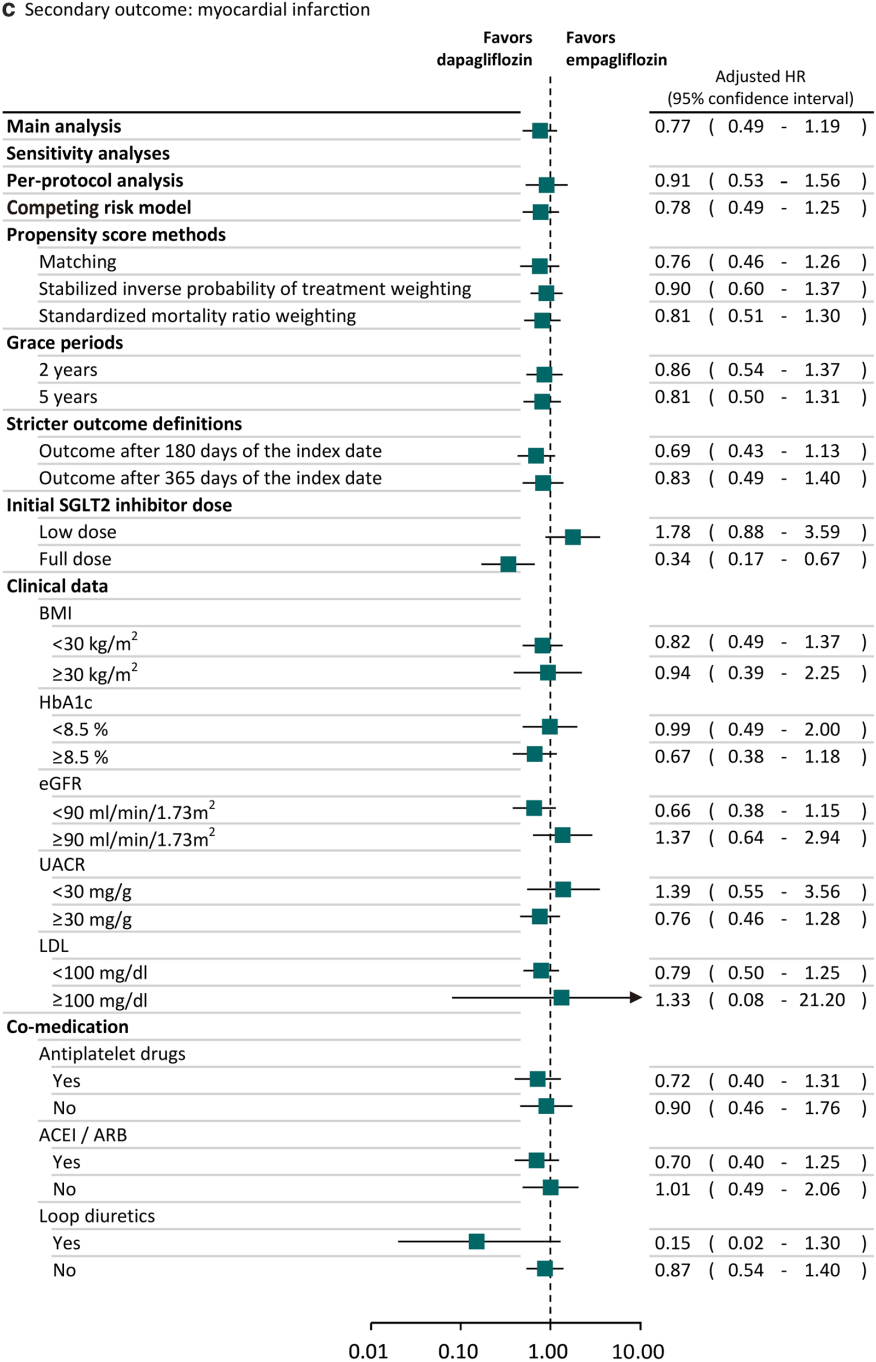

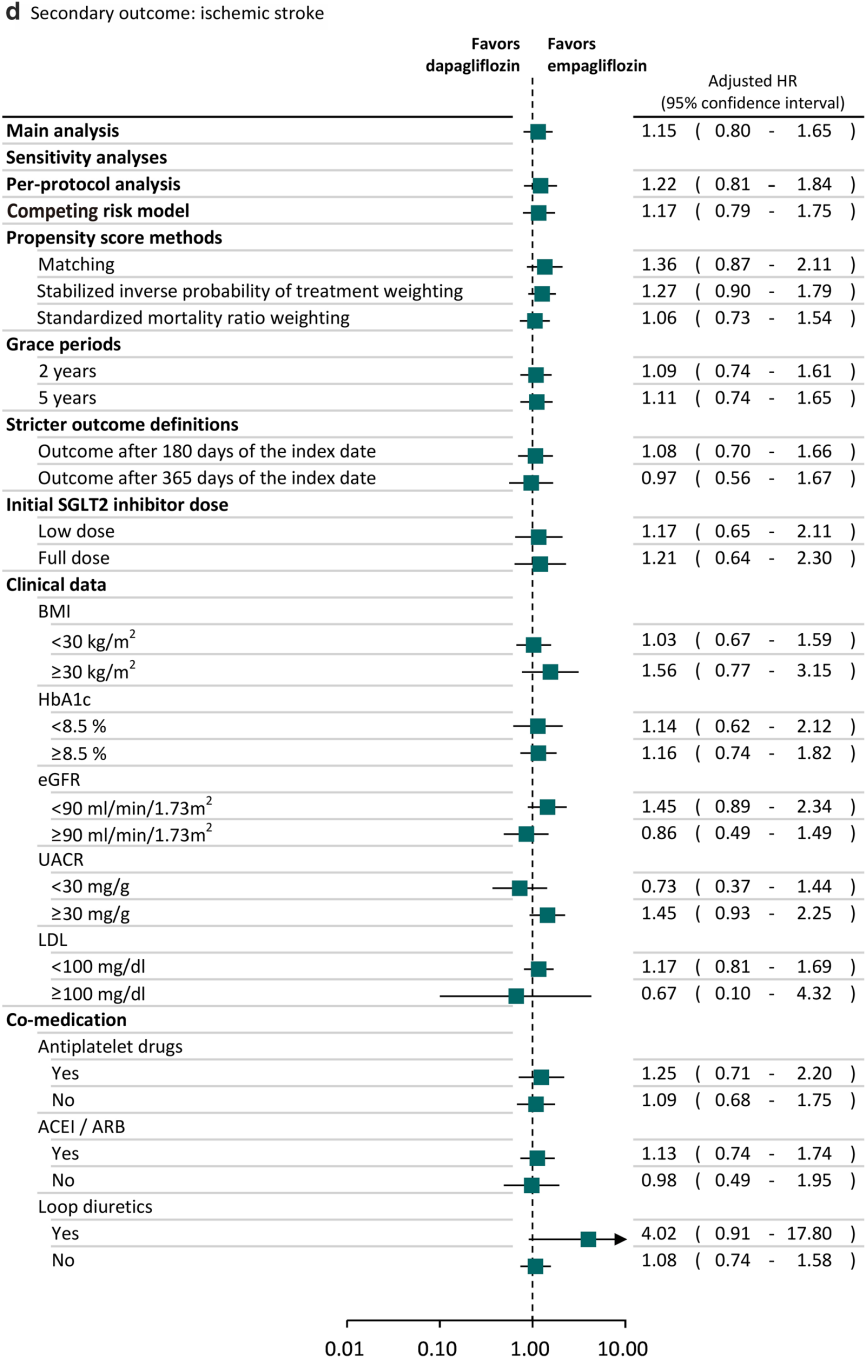

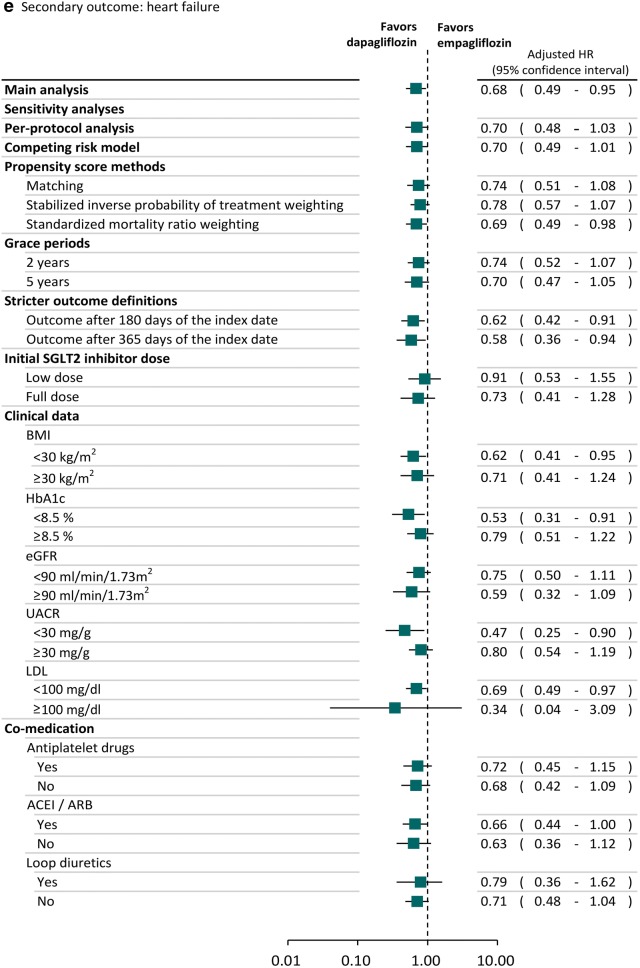
Sensitivity and subgroup analyses of comparative risk for cardiovascular events between SGLT2 inhibitors. *BMI* body mass index, *CI* confidence interval, *eGFR* estimated glomerular filtration rate, *HR* hazard ratio, *LDL* low-density lipoprotein, *SIPTW* stabilized inverse probability of treatment weighting, *SMRW* standardized mortality ratio weighting, *UACR* urine albumin–creatinine ratio

The trends of results from sensitivity and subgroup analyses were consistent with the main analysis (Fig. 2). Specifically, we found the lower risk of heart failure associated with dapagliflozin was also statistically significant in several sensitivity analyses. For example, the observed effects were still found after we used SMRW analyses (adjusted HR: 0.69; 95% CI 0.49–0.98). We found the results of sensitivity analyses in the subgroups of patients receiving low SGLT2 inhibitor dose (adjusted HR: 0.91; 95% CI 0.53–1.55) or full SGLT2 inhibitor dose (adjusted HR: 0.73; 95% CI 0.41–1.28) and different baseline cardiovascular risks based on the clinical data, such as BMI ≥ 30 kg/m^2^ (adjusted HR: 0.71; 95% CI 0.41–1.24) or BMI < 30 kg/m^2^ (adjusted HR: 0.62; 95% CI 0.41–0.95), were consistent with the main analysis, in that patients receiving dapagliflozin had lower risk of heart failure compared to empagliflozin. Dapagliflozin reduced the risk of heart failure compared to empagliflozin regardless of whether or not anti-platelet drugs, ACEI/ARB and loop diuretics were used concomitantly.

## Discussion

Clinical trials have demonstrated the favorable cardiovascular effects of empagliflozin and dapagliflozin [32]. In the EMPA-REG OUTCOME trial, empagliflozin showed 14% reduction of composite cardiovascular events and 35% reduction of heart failure compared to placebo. In the DECLARE-TIMI 58 trials, dapagliflozin lowered the risk by 27% for heart failure compared to placebo [33, 34]. Notably, the protective effects in regard to heart failure remained robust after several post hoc analyses from the EMPA-REG OUTCOME and DECLARE-TIMI 58 trials [35–37]. However, differences in patient phenotypes in clinical trials and in drug properties between SGLT2 inhibitors may have produced varied cardiovascular effects [38], so this study could provide additional information about the comparative evaluations associated with dapagliflozin vs. empagliflozin.

The incidence rates of cardiovascular events in patients newly receiving dapagliflozin and empagliflozin were 12.3 and 16.3 per 1000 person-years in this real-world cohort. We found a higher incidence of cardiovascular events in empagliflozin than dapagliflozin in the present study as compared to that among clinical trials (13.4–15.8 per 1000 person-years) [32]. Possible reasons may involve the channeling of SGLT2 inhibitor use in clinical practice based on medical evidence. First, the cardiovascular benefits of empagliflozin were discovered earlier in clinical trials than those of dapagliflozin [33, 34], so clinical physicians might have prescribed empagliflozin to type 2 diabetes patients with high risk of cardiovascular diseases (e.g., more cardiovascular co-morbidity and concomitant cardiovascular mediation) at baseline. Second, empagliflozin could be initiated in patients with eGFR > 45 mL/min/1.73 m^2^, while dapagliflozin was only recommended in patients with eGFR > 60 mL/min/1.73 m^2^ [39]. It is possible that some empagliflozin users with impaired renal functions contributed to a higher baseline cardiovascular disease risk in this group [40]. After adjusting for these confounders by regression models, we found the initiation of dapagliflozin was associated with a similar risk of composite cardiovascular events as compared to empagliflozin.

An excess heart failure risk persists in type 2 diabetes patients despite optimal control of an array of conventional risk factors, including hyperglycaemia [41]. To date, only SGLT2 inhibitors have produced a robust and significant reduction of heart failure risk, which has remained of similar magnitude regardless of a history of heart failure or established cardiovascular diseases [24, 25, 42, 43]. In addition to the favorable effects on many co-morbidities related to heart failure, including diabetes, obesity and hypertension, the sodium excretion and osmotic diuresis associated with SGLT2 inhibitors could also contribute to a reduced risk of heart failure [44–46].

The incidence of heart failure in this study was 4.9 and 9.0 per 1000 patient-years in the dapagliflozin and empagliflozin groups, respectively. Based on the post hoc analysis from 97 heart failure cases followed by the initiations of the SGLT2 inhibitor treatment from 3 Chang Gung Memorial hospitals, we found 67.0% vs. 28.9% of them were heart failure with preserved ejection fraction (HFpEF, LVEF ≥ 40%) and heart failure with reduced ejection fraction (HFrEF, LVEF < 40%) respectively, and 4.1% were without a report of LVEF. We determined the outcome of heart failure by all hospital events from inpatient and outpatient records, because heart failure in real-world patients with type 2 diabetes often remains undiagnosed, and, if present, sharply increases mortality risk [25]. Our estimates were similar to the reports of heart failure with SGLT2 inhibitors in clinical trials (7.0–8.9 per 1000 patient-years) [32].

The possible explanations for the reduction of heart failure in dapagliflozin and empagliflozin have been addressed. For example, previous studies indicated dapagliflozin has beneficial effects on left ventricular diastolic functions, vascular remodeling and cardiometabolic markers [47–49]. Empagliflozin has also been reported with salutary changes in left ventricular mass and diastolic function in type 2 diabetes patients [50], which might decrease the risk of heart failure. Based on evidence from previous trials and our analyses, although both SGLT2 inhibitors seem to reduce the risk of heart failure, we consider that dapagliflozin may have greater effects on heart failure reduction compared to empagliflozin. A possible explanation of this finding may lie in the differences in the drugs’ pharmacokinetic properties and in SGLT2/SGLT1 receptor selectivity. First, dapagliflozin has longer lasting pharmacological effects [13], such as sodium excretion and osmotic dieresis [14, 15], and it may reduce blood pressure variability, which is beneficial with regard to heart failure [16, 17]. In addition, it has been reported that the ratio of SGLT2:SGLT1 receptor selectivity is lower in dapagliflozin (1200-fold) than in empagliflozin (2500-fold) [51]. Previous studies have indicated SGLT1 receptors are predominantly in the human intestine and the higher selectivity of SGLT1 receptors can lower the variations of postprandial blood glucose, which might help to reduce heart failure risk [52–54]. Although some reports indicated SGLT1 receptors are also expressed in cardiac myocytes which may reduce cardiac functions, the findings remain controversial because of conflicting conclusions from the studies [33, 47–49, 55]. Future studies are required to confirm the mechanisms addressed above to account for the difference in reduction of heart failure risks between SGLT2 inhibitors.

The risk of ischemic stroke in patients with diabetes mellitus is increased twofold compared with individuals without diabetes mellitus [1]. Therefore, it could be of great value to find out if different anti-diabetes medications have any protective or harmful effects regarding stroke, and compare them. Meta-analyses of randomized controlled trials showed no significant differences in stroke risk among different SGLT2 inhibitors [56]. Among SGLT2 inhibitors, we found a trend, though not significant, towards increased risk of ischemic stroke for dapagliflozin compared to empagliflozin in this study. It has been reported that the use of glucagon-like peptide-1 receptor agonists (GLP-RA) reduced the risk of stroke [57, 58], and therefore the significantly higher proportion of GLP1-RA comediation in the empagliflozin group might have contributed to the lower risk of stroke. Although the use of GLP1-RA has been considered in the multivariate Cox regression models, we also conducted a post hoc analysis to exclude patients receiving GLP1-RA and re-examined the risk of ischemic stroke. The result of this post hoc analysis (adjusted HR: 1.19; 95% CI 0.82–1.72) remained consistent with the main analysis. Concerns have been raised that elevated hematocrit and hypotension after the SGLT2 inhibitor treatment may be associated with an increased risk of stroke caused by sludging and hypoperfusion, respectively. Empagliflozin and dapagliflozin have been reported to increase hematocrit in clinical trials [59, 60], but little has been known about the comparative effects of hematocrit changes. However, several meta-analyses have shown no clinical difference in the reductions of systolic and diastolic blood pressures between empagliflozin and dapagliflozin [11, 61]. Considering that different patients’ characteristics in previous clinical trials based on the various inclusion and exclusion criteria could affect stroke risk, further head-to-head studies should confirm this finding.

Strengths of this study included the large real-world cohort to compare the cardiovascular risk in type 2 diabetes patients between dapagliflozin and empagliflozin. Additionally, this study included laboratory measurements which describe important contributing factors for cardiovascular diseases but which are usually lacking in most other administrative databases and which can allow a more precise estimate of cardiovascular risk. Finally, the consistent findings across sensitivity analyses support our internal validity.

We acknowledge some potential limitations in this retrospective cohort study. First, our findings cannot be applied to the type 2 diabetes patients with major cardiovascular diseases at baseline because we focused on new cardiovascular events. Furthermore, our findings are derived from statistical inferences based on the use of several models to deal with baseline imbalances between groups. Future head-to-head prospective studies should confirm our findings. Third, information on some potential unmeasured confounders is not available in the CGRD, and these factors may have confounded the observed association between SGLT2 inhibitors and cardiovascular events. However, a neutral risk association of atrial fibrillation (negative control outcome) was observed between dapagliflozin and empagliflozin, which is indicative of a balanced profile for residual confounding and bias due to unobserved confounders at baseline. Fourth, while selection bias may be present in this study, we used several propensity score testing approaches to address observed potential confounders using as many as 40 covariates. Fifth, the competing risk of mortality may either hinder or modify the observation of cardiovascular events, and hence we used the Fine and Gray sub-distribution hazards model, which revealed similar risks as the main analyses. Sixth, we did not obtain data from outside the CGRD in Taiwan, which may have resulted in loss to follow-up. However, the findings from the per-protocol approach to minimize the effects of loss to follow-up were consistent with the main analyses. Seventh, we did not have information on diabetes duration, but we included patients’ laboratory data (e.g., glycemic and renal parameters) which are important determinants of cardiovascular risk. Finally, the comparisons associated with dapagliflozin vs. empagliflozin in these analyses apply only to cardiovascular outcomes, and no inference on side effects can be made from this study. We conducted a post hoc investigation of the urinary tract infection rate, which is the most commonly reported adverse effect of SGLT2 inhibitors, and found 7.7 and 7.5 per 1000 person-years in dapagliflozin and empagliflozin, respectively. We considered potential confounding factors in the regression models for adjustment and conducted a series of sensitivity and validation analyses to confirm the findings; however, some aforementioned limitations of the study remained unavoidable. Notwithstanding, the findings from this study may provide a clinical hypothesis for future prospective studies to confirm the differences in cardiovascular outcomes between SGLT2 inhibitors.

## Conclusions

In this real-world study, we did not find a significant difference between dapagliflozin and empagliflozin in the risk of cardiovascular events among type 2 diabetes patients. The results showed that dapagliflozin users had a significantly lower risk of heart failure as compared to empagliflozin users, whereby the exact mechanisms of this difference require further studies.

## Supplementary information

**Additional file 1. Table S1.** Diagnosis code for study outcome and co-morbidity.

**Additional file 2. Table S2.** Individual drug for study co-medication.

**Additional file 3. Table S3.** Baseline patient characteristics before and after propensity score methods.

**Additional file 4. Table S4.** Falsification analysis presenting hazard ratios for incident atrial fibrillation between SGLT2 inhibitors.

## Data Availability

Data sharing is not applicable to this study as data management and analysis were performed on a statistics server through remote access in Chang Gung Medical Foundation in Taiwan, for privacy and safety concerns.

## Abbreviations

BMI: body mass index
CGRD: Chang Gung Research Database
CI: confidence intervals
DPP-4: dipeptidyl peptidase 4
eGFR: estimated glomerular filtration rate
GLP-1: glucagon-like-peptide 1
HR: hazard ratios
ICD-9-CM: International Classification of Diseases, Ninth Revision, Clinical Modification
LDL: low-density lipoprotein
SGLT2: sodium–glucose co-transporter 2
SIPTW: stabilized inverse probability of treatment weighting
SMRW: standardized mortality ratio weighting
UACR: urine albumin–creatinine ratio
