# Supplementary material for: Comparative risk evaluation for cardiovascular events associated with dapagliflozin vs. empagliflozin in real-world type 2 diabetes patients: a multi-institutional cohort study
Source: Cardiovasc Diabetol. 2019 Sep 24;18:120. doi: 10.1186/s12933-019-0919-9 (PMC6760106; doi:10.1186/s12933-019-0919-9)
Supplement: Supplementary file 1 — Additional file 1. Table S1. Diagnosis code for study outcome and co-morbidity. [file 12933_2019_919_MOESM1_ESM.docx]

| Table S1. Diagnosis code for study outcome and co-morbidity | | |
| --- | --- | --- |
| Diseases | ICD-9-CM codes | ICD-10-CM codes |
| Outcome |  |  |
| Cardiovascular mortality | 393-398, 401-405, 410-414, 415-417, 420-429, 430-438, 440-448, 451-459 | I05-I09, I10-I15, I20-I25, I26-I28, I30-I32, I60-I69, I70-I79, I80-I89, I95-I99 |
| Myocardial infarction | 410 | I21 |
| Ischemic stroke | 433, 434 | I63 |
| Heart failure | 428 | I50 |
| Comorbidity |  |  |
| Hypertension | 401, 402, 403, 404, 405 | I10, I11, I12, I13, I15, I16 |
| Hyperlipidemia | 272 | E78 |
| Coronary heart disease* | 411, 412, 413, 414 | I20, I22, I23, I24, I25 |
| Atrial fibrillation | 42731 | I48 |
| Peripheral artery disease | 440 | I70, I73 |
| Diabetic retinopathy | 2505 | E083, E113 |
| Diabetic neuropathy | 2506 | E084, E114 |
| Diabetic nephropathy | 2504 | E082, E112 |
| Chronic obstructive pulmonary disease | 491, 492, 496 | J44 |
| Liver disease | 456, 571, 572 | I85, K70, K71, K72, K73, K74, K75, K76, K77 |
| Depression | 2962, 2963 | F32, F33 |
| Schizophrenia | 295 | F20 |
| Cancer | 140-239 | C0-D4 |
| *Coronary heart diseases did not include myocardial infarction (ICD-9: 410; ICD-10: I21) because prevalent myocardial infarction cases were excluded before initiation of SGLT2 inhibitors in this study. | | |
