# Supplementary material for: Comparative risk evaluation for cardiovascular events associated with dapagliflozin vs. empagliflozin in real-world type 2 diabetes patients: a multi-institutional cohort study
Source: Cardiovasc Diabetol. 2019 Sep 24;18:120. doi: 10.1186/s12933-019-0919-9 (PMC6760106; doi:10.1186/s12933-019-0919-9)
Supplement: Supplementary file 2 — Additional file 2. Table S2. Individual drug for study co-medication. [file 12933_2019_919_MOESM2_ESM.docx]

| Table S2. Individual drug for study co-medication | |
| --- | --- |
| Drug class | Drug name |
| Anti-platelet agents | Aspirin, Clopidogrel, Dipyridamole, Ticagrelor |
| Anti-coagulant agents | Warfarin, Apixaban, Dabigatran, Edoxaban, Rivaroxaban |
| Beta blocker | Atenolol, Bisoprolol, Carvedilol, Metoprolol, Propranolol, |
| Angiotensin-converting enzyme inhibitors or angiotensin receptor blockers | Azilsartan, Candestartan, Captopril, Enalapril, Fosinopril, Irbesartan, Losartan, Olmesartan, Ramipril Telmisartan, Valsartan |
| Calcium channel blockers | Amlodipine, Diltiazem, Felodipine, Lercanidipine, Nifedipine, Verapamil |
| Diuretics | Acetazolamide, Amiloride, Benzyl hydrochlorothiazide, Bumetanide, Eplerenone**,** Furosemide, Hydrochlorothiazide, Indapamide, Spironolactone |
| Loop diuretics | Bumetanide, Furosemide |
| Thiazides | Amiloride, Benzyl hydrochlorothiazide, Hydrochlorothiazide, Indapamide |
| Mineralocorticoid receptor antagonist | Spironolactone |
| Statin | Atorvastatin, Fluvastatin, Pitavastatin, Rosuvastatin, Simvastatin |
| Fibrate | Fenofibrate, Gemfibrozil |
| Sulfonylurea | Glipizide, Gliclazide, Glimepiride, Glyburide |
| Dipeptidyl peptidase-4 inhibitors | Alogliptin, Linagliptin, Saxagliptin, Sitagliptin, Vidagliptin |
| Alpha-glucosidase inhibitors | Acarbose |
| Glinides | Repaglinide, Meglitinide, Nateglinide |
| Thiazolidinediones | Pioglitzone |
| Glucagon-like peptide-1 receptor antagonist | Dulaglutide, Exenatide, Liraglutide |
| Insulin | Rapid, short, intermediate and long-acting insulins |
| NSAID | Acemetacin, Celecoxib, Diclofenac, Indomethacin, Ibuprofen, Meloxicam, Naproxen, Sulindac |
