# Supplementary material for: Comparative risk evaluation for cardiovascular events associated with dapagliflozin vs. empagliflozin in real-world type 2 diabetes patients: a multi-institutional cohort study
Source: Cardiovasc Diabetol. 2019 Sep 24;18:120. doi: 10.1186/s12933-019-0919-9 (PMC6760106; doi:10.1186/s12933-019-0919-9)
Supplement: Supplementary file 3 — Additional file 3. Table S3. Baseline patient characteristics before and after propensity score methods. [file 12933_2019_919_MOESM3_ESM.docx]

| Table S3. Baseline patient characteristics before and after propensity score methods | | | | | | | | | | | | |
| --- | --- | --- | --- | --- | --- | --- | --- | --- | --- | --- | --- | --- |
|  | Before propensity score methods | | | After propensity score matching | | | After SIPTW | | | After SMRW | | |
|  | Dapagliflozin | Empagliflozin | ASMD^a^ | Dapagliflozin | Empagliflozin | ASMD^a^ | Dapagliflozin | Empagliflozin | ASMD^a^ | Dapagliflozin | Empagliflozin | ASMD^a^ |
| Patients, % | 5812 | 6869 |  | 4432 | 4432 |  | 5745 | 6964 |  | 5812 | 5989 |  |
| Age, % |  |  |  |  |  |  |  |  |  |  |  |  |
| <65 | 69.5 | 65.5 | 0.09 | 68.2 | 68.3 | 0.00 | 67.1 | 67.3 | 0.00 | 69.5 | 69.4 | 0.00 |
| ≧65 | 30.5 | 34.5 | 0.09 | 31.8 | 31.7 | 0.00 | 32.9 | 32.7 | 0.00 | 30.5 | 30.6 | 0.00 |
| Female, % | 45.5 | 42.5 | 0.06 | 45.1 | 45.7 | 0.01 | 44.3 | 44.2 | 0.00 | 45.5 | 46.1 | 0.01 |
| PDD/DDD, mean | 0.9 | 0.8 | 0.31 | 0.9 | 0.8 | 0.03 | 0.9 | 0.9 | 0.02 | 0.9 | 0.9 | 0.21* |
| BMI,% |  |  |  |  |  |  |  |  |  |  |  |  |
| <30 | 67.2 | 66.7 | 0.01 | 67.5 | 66.2 | 0.03 | 67.3 | 66.2 | 0.02 | 67.2 | 65.7 | 0.03 |
| ≥30 | 32.8 | 33.3 | 0.01 | 32.5 | 33.8 | 0.03 | 32.7 | 33.8 | 0.02 | 32.8 | 34.3 | 0.03 |
| HbA1c, % |  |  |  |  |  |  |  |  |  |  |  |  |
| <8.5 | 43.7 | 48.2 | 0.09 | 46.7 | 45.0 | 0.03 | 46.2 | 46.0 | 0.01 | 43.7 | 43.5 | 0.01 |
| ‎≥8.5 | 56.3 | 51.8 | 0.09 | 53.3 | 55.0 | 0.03 | 53.8 | 54.0 | 0.01 | 56.3 | 56.5 | 0.01 |
| eGFR, % |  |  |  |  |  |  |  |  |  |  |  |  |
| <60 | 6.0 | 13.6 | 0.26 | 7.8 | 7.1 | 0.03 | 10.4 | 10.1 | 0.01 | 6.0 | 6.1 | 0.00 |
| 60-90 | 36.2 | 36.0 | 0.00 | 36.8 | 36.9 | 0.00 | 36.0 | 36.2 | 0.01 | 36.2 | 36.5 | 0.01 |
| ‎≥90 | 57.9 | 50.5 | 0.15 | 55.4 | 56.0 | 0.01 | 53.7 | 53.7 | 0.00 | 57.9 | 57.4 | 0.01 |
| UACR, % |  |  |  |  |  |  |  |  |  |  |  |  |
| <30 mg/g | 51.7 | 48.6 | 0.06 | 51.2 | 50.2 | 0.02 | 50.7 | 49.3 | 0.03 | 51.7 | 50.0 | 0.03 |
| 30-300 mg/g | 31.6 | 32.0 | 0.01 | 31.5 | 31.5 | 0.00 | 31.7 | 32.0 | 0.01 | 31.6 | 32.0 | 0.01 |
| ‎≥300 mg/g | 16.7 | 19.4 | 0.07 | 17.3 | 18.3 | 0.03 | 17.6 | 18.7 | 0.03 | 16.7 | 18.0 | 0.03 |
| LDL, % |  |  |  |  |  |  |  |  |  |  |  |  |
| ‎≥100 mg/dl | 1.8 | 2.0 | 0.01 | 1.8 | 2.10 | 0.02 | 1.8 | 2.0 | 0.01 | 1.8 | 2.0 | 0.02 |
| <100 mg/dl | 98.2 | 98.0 | 0.01 | 98.2 | 97.9 | 0.02 | 98.2 | 98.0 | 0.01 | 98.2 | 98.0 | 0.02 |
| Index year,% |  |  |  |  |  |  |  |  |  |  |  |  |
| 2016 | 57.2 | 52.8 | 0.09 | 54.6 | 55.3 | 0.01 | 54.0 | 53.7 | 0.00 | 57.2 | 54.9 | 0.05 |
| 2017 | 42.8 | 47.2 | 0.09 | 45.4 | 44.7 | 0.01 | 46.0 | 46.3 | 0.00 | 42.8 | 45.1 | 0.05 |
| Hospital level, % |  |  |  |  |  |  |  |  |  |  |  |  |
| Medical centers | 55.5 | 57.2 | 0.03 | 54.7 | 55.3 | 0.01 | 56.4 | 56.3 | 0.00 | 55.5 | 55.4 | 0.00 |
| Regional hospitals | 26.5 | 22.3 | 0.10 | 26.3 | 25.1 | 0.03 | 24.1 | 24.2 | 0.00 | 26.5 | 26.4 | 0.00 |
| District hospitals | 18.0 | 20.5 | 0.06 | 19.0 | 19.5 | 0.02 | 19.5 | 19.5 | 0.00 | 18.0 | 18.2 | 0.01 |
| Department, % |  |  |  |  |  |  |  |  |  |  |  |  |
| Metabolism & Endocrinology | 68.2 | 61.2 | 0.15 | 65.4 | 66.2 | 0.02 | 64.2 | 64.5 | 0.01 | 68.2 | 68.2 | 0.00 |
| Cardiology | 20.3 | 26.1 | 0.14 | 22.7 | 20.9 | 0.04 | 23.7 | 23.3 | 0.01 | 20.3 | 20.1 | 0.01 |
| Others | 11.5 | 12.7 | 0.04 | 11.9 | 12.9 | 0.03 | 12.2 | 12.2 | 0.00 | 11.5 | 11.7 | 0.01 |
| Comorbidity, % |  |  |  |  |  |  |  |  |  |  |  |  |
| Hypertension | 64.0 | 67.1 | 0.06 | 65.7 | 64.8 | 0.02 | 66.0 | 65.8 | 0.00 | 64.0 | 64.3 | 0.01 |
| Hyperlipidemia | 73.5 | 72.4 | 0.03 | 73.5 | 71.9 | 0.04 | 73.7 | 72.7 | 0.02 | 73.5 | 73.1 | 0.01 |
| Coronary heart disease^b^ | 14.3 | 17.6 | 0.09 | 15.5 | 14.3 | 0.03 | 15.8 | 16.1 | 0.01 | 14.3 | 14.4 | 0.00 |
| Atrial fibrillation | 1.9 | 2.4 | 0.03 | 2.3 | 1.9 | 0.03 | 2.3 | 2.1 | 0.02 | 1.9 | 1.7 | 0.02 |
| Peripheral artery disease | 1.2 | 1.6 | 0.04 | 1.2 | 1.3 | 0.01 | 1.3 | 1.5 | 0.02 | 1.2 | 1.4 | 0.02 |
| Diabetic retinopathy | 8.2 | 8.4 | 0.01 | 8.5 | 8.3 | 0.01 | 8.5 | 8.6 | 0.00 | 8.2 | 8.5 | 0.01 |
| Diabetic neuropathy | 10.0 | 9.1 | 0.04 | 9.5 | 9.7 | 0.04 | 9.9 | 9.1 | 0.02 | 10.0 | 9.0 | 0.03 |
| Diabetic nephropathy | 26.0 | 27.6 | 0.03 | 25.8 | 27.5 | 0.01 | 26.4 | 27.5 | 0.03 | 26.0 | 27.3 | 0.04 |
| Chronic obstructive pulmonary disease | 2.2 | 2.6 | 0.03 | 2.4 | 2.4 | 0.00 | 2.3 | 2.4 | 0.00 | 2.2 | 2.2 | 0.00 |
| Liver disease | 18.1 | 18.6 | 0.01 | 18.1 | 19.6 | 0.04 | 17.9 | 18.9 | 0.03 | 18.1 | 19.3 | 0.03 |
| Depression | 1.5 | 1.5 | 0.00 | 1.6 | 1.5 | 0.01 | 1.5 | 1.5 | 0.00 | 1.5 | 1.5 | 0.01 |
| Schizophrenia | 0.4 | 0.3 | 0.02 | 0.5 | 0.4 | 0.01 | 0.4 | 0.3 | 0.01 | 0.4 | 0.4 | 0.00 |
| Cancer | 6.3 | 6.5 | 0.01 | 6.5 | 6.3 | 0.01 | 6.3 | 6.4 | 0.00 | 6.3 | 6.3 | 0.00 |
| [Charlson comorbidity index score](https://www.mdcalc.com/charlson-comorbidity-index-cci), % |  |  |  |  |  |  |  |  |  |  |  |  |
| <2 | 42.0 | 39.6 | 0.05 | 41.8 | 40.3 | 0.03 | 40.9 | 40.2 | 0.01 | 42.0 | 40.9 | 0.02 |
| ‎≥2 | 58.0 | 60.4 | 0.05 | 58.2 | 59.8 | 0.03 | 59.1 | 59.8 | 0.01 | 58.0 | 59.1 | 0.02 |
| Previous hospitalization, % | 9.5 | 11.9 | 0.08 | 11.1 | 11.2 | 0.00 | 11.2 | 10.9 | 0.01 | 9.5 | 9.7 | 0.01 |
| Concomitant medications, % |  |  |  |  |  |  |  |  |  |  |  |  |
| Anti-platelet agents | 26.7 | 30.2 | 0.08 | 28.6 | 27.9 | 0.02 | 29.1 | 28.9 | 0.01 | 26.7 | 27.4 | 0.02 |
| Anti-coagulant agents | 1.7 | 2.2 | 0.03 | 1.9 | 1.7 | 0.02 | 2.0 | 1.9 | 0.01 | 1.7 | 1.5 | 0.02 |
| Beta blockers | 21.5 | 24.6 | 0.07 | 22.9 | 22.3 | 0.02 | 23.6 | 23.1 | 0.01 | 21.5 | 21.4 | 0.00 |
| Angiotensin-converting enzyme inhibitors or angiotensin receptor blockers | 55.7 | 60.1 | 0.09 | 57.2 | 56.8 | 0.01 | 58.0 | 58.5 | 0.01 | 55.7 | 56.7 | 0.02 |
| Calcium channel blockers | 35.4 | 40.4 | 0.10 | 36.8 | 37.5 | 0.01 | 37.5 | 38.8 | 0.03 | 35.4 | 37.0 | 0.04 |
| Loop diuretics | 2.6 | 4.1 | 0.08 | 3.1 | 3.6 | 0.03 | 3.4 | 3.7 | 0.01 | 2.6 | 3.1 | 0.03 |
| Thiazides | 4.7 | 5.0 | 0.02 | 5.0 | 4.8 | 0.01 | 4.9 | 4.8 | 0.01 | 4.7 | 4.5 | 0.01 |
| Mineralocorticoid receptor antagonist | 1.0 | 1.8 | 0.07 | 1.3 | 1.4 | 0.01 | 1.6 | 1.5 | 0.01 | 1.0 | 1.1 | 0.01 |
| Statin | 63.0 | 66.1 | 0.06 | 63.9 | 62.4 | 0.03 | 64.6 | 64.8 | 0.00 | 63.0 | 63.3 | 0.01 |
| Fibrate | 9.2 | 10.4 | 0.04 | 9.7 | 10.3 | 0.02 | 10.0 | 9.9 | 0.01 | 9.2 | 9.2 | 0.00 |
| Ezetimibe | 11.1 | 11.5 | 0.01 | 11.4 | 11.4 | 0.00 | 11.4 | 11.5 | 0.00 | 11.1 | 11.4 | 0.01 |
| Metformin | 92.3 | 89.2 | 0.11 | 90.9 | 91.3 | 0.01 | 90.5 | 90.6 | 0.00 | 92.3 | 92.3 | 0.00 |
| Sulfonylurea | 62.0 | 54.6 | 0.15 | 58.6 | 59.8 | 0.03 | 58.0 | 58.2 | 0.01 | 62.0 | 62.4 | 0.01 |
| Dipeptidyl peptidase-4 inhibitors | 62.2 | 64.6 | 0.05 | 61.3 | 63.2 | 0.04 | 63.4 | 63.4 | 0.00 | 62.2 | 62.1 | 0.00 |
| Alpha-glucosidase inhibitors | 17.2 | 18.1 | 0.02 | 16.9 | 17.4 | 0.01 | 17.8 | 17.8 | 0.00 | 17.2 | 17.4 | 0.01 |
| Glinides | 1.6 | 2.3 | 0.05 | 1.7 | 1.9 | 0.02 | 1.9 | 2.2 | 0.02 | 1.6 | 2.1 | 0.03 |
| Thiazolidinediones | 26.7 | 23.1 | 0.08 | 26.7 | 25.8 | 0.02 | 24.9 | 24.9 | 0.00 | 26.7 | 27.0 | 0.01 |
| Glucagon-like peptide-1 receptors antagonist | 1.3 | 2.3 | 0.07 | 1.7 | 1.5 | 0.01 | 1.8 | 1.8 | 0.00 | 1.3 | 1.3 | 0.00 |
| Insulin | 18.6 | 21.9 | 0.08 | 20.9 | 20.9 | 0.00 | 20.4 | 20.3 | 0.00 | 18.6 | 18.4 | 0.01 |
| Non-steroidal anti-inflammatory drugs | 8.2 | 7.9 | 0.01 | 8.8 | 7.4 | 0.05 | 8.5 | 7.8 | 0.03 | 8.2 | 7.6 | 0.02 |
| ASMD, absolute standardized mean difference; BMI, body mass index; DDD, defined daily dose; eGFR, estimated glomerular filtration rate; LDL, low-density lipoprotein; PDD,  prescribed daily dose; UACR, urine albumin-creatinine ratio  ^a^ We used ASMD to assess the balance of baseline covariates between dapagliflozin and empagliflozin groups, and an ASMD <0.1 indicates a negligible difference in potential confounders between study groups [1]. We used the multivariate Cox regression model to adjust the variables with ASMD≥0.1 after propensity score methods.  ^b^ Myocardial infarction was not included because prevalent myocardial infarction cases were excluded before initiation of SGLT2 inhibitors in this study. | | | | | | | | | | | | |

Reference

1. Austin PC. The use of propensity score methods with survival or time-to-event outcomes: reporting measures of effect similar to those used in randomized experiments. Stat Med 2014;33: 1242–58.
