# Supplementary material for: Comparative risk evaluation for cardiovascular events associated with dapagliflozin vs. empagliflozin in real-world type 2 diabetes patients: a multi-institutional cohort study
Source: Cardiovasc Diabetol. 2019 Sep 24;18:120. doi: 10.1186/s12933-019-0919-9 (PMC6760106; doi:10.1186/s12933-019-0919-9)
Supplement: Supplementary file 4 — Additional file 4. Table S4. Falsification analysis presenting hazard ratios for incident atrial fibrillation between SGLT2 inhibitors. [file 12933_2019_919_MOESM4_ESM.docx]

| Table S4. Falsification analysis presenting hazard ratios for incident atrial fibrillation between SGLT2 inhibitors. | | | |
| --- | --- | --- | --- |
|  | Atrial fibrillation | | |
|  | Incidence rate | Crude HR (95% CI) | Adjusted HR (95% CI) |
| Empagliflozin (n=7,591) | 3.5 / 1,000 person-years | Reference | Reference |
| Dapagliflozin  (n=6,299) | 3.3 / 1,000 person-years | 0.92 (0.62-1.36) | 1.08 (0.73-1.60) |
| 1. Multivariate Cox proportional hazards models were used to estimate the HR of atrial fibrillation outcomes and were adjusted for baseline variables as in Table 1, the history of myocardial infarction, ischemic stroke and heart failure that were significantly related to outcomes from the prior uni-variate Cox regression model at alpha level of 0.1. 2. This modeling approach is used to mitigate potential selection bias due to unmeasured factors. | | | |
